# The p-ERG spatial acuity in the biomedical pig under physiological conditions

**DOI:** 10.1038/s41598-022-19925-8

**Published:** 2022-09-14

**Authors:** Domenico Ventrella, José Fernando Maya-Vetencourt, Alberto Elmi, Francesca Barone, Camilla Aniballi, Luisa Vera Muscatello, Maurizio Mete, Grazia Pertile, Fabio Benfenati, Maria Laura Bacci

**Affiliations:** 1 Department of Veterinary Medicine, Alma Mater Studiorum-University of Bologna, Ozzano dell’Emilia, BO Italy; 2 Department of Biology, University of Pisa, Pisa, PI Italy; 3 Center for Synaptic Neuroscience and Technology, Italian Institute of Technology IIT, Genoa, GE Italy; 4 Unit on Ocular Stem Cell and Translational Research, National Eye Institute (NEI), Bethesda, MD USA; 5 IRCCS Sacro Cuore Don Calabria Hospital, Negrar, VR Italy; 6 IRCCS San Martino Hospital, Genoa, GE Italy

**Keywords:** Neurophysiology, Translational research

## Abstract

Pigs are becoming an important pre-clinical animal species for translational ophthalmology, due to similarities with humans in anatomical and physiological patterns. Different models of eye disorders have been proposed, and they are good candidates to assess biocompatibility/functionality of retinal prostheses. Electroretinography is a common tool allowing to gain information on retinal function, with several types of electroretinogram (ERG) been implemented including full field (ff-ERG), multifocal (mf-ERG) and pattern (p-ERG). p-ERG represents a valuable tool to monitor Retinal Ganglion Cells (RGCs) activity and can be used to calculate p-ERG spatial acuity. Unfortunately, scarce methodological data are available regarding recording/interpretation of p-ERG and retinal acuity in biomedical pigs yet enhancing knowledge regarding pig vision physiology will allow for more refined and responsible use of such species. Aim of this study was to record p-ERG in juvenile pigs to functionally assess visual acuity. Six female hybrid pigs underwent two p-ERG recording sessions at 16 and 19 weeks of age. Photopic ff-ERG were also recorded; optical coherence tomography (OCT) and histology were used to confirm retinal integrity. ff-ERG signals were repeatable within/across sessions. All p-ERG traces consistently displayed characterizing peaks, and the progressive decrease of amplitude in response to the increment of spatial frequency revealed the reliability of the method. Mean p-ERG spatial acuities were 5.7 ± 0.14 (16 weeks) and 6.2 ± 0.15 cpd (19 weeks). Overall, the p-ERG recordings described in the present work seem reliable and repeatable, and may represent an important tool when it comes to vision assessment in pigs.

## Introduction

When looking at relevant ophthalmologic animal models for biomedical purposes, the pig has gained a lot of ground in the last years, due to the similarities with humans in physiological and genomic patterns, and the great correlation in size and anatomy^1–4^. The swine retina contains a cone-dominant central area, known as the visual streak, with a rod-enriched peripheral retina^5–7^. Out of the most used large preclinical species, the choice of the pig seems to be more acceptable than non-human primates (NHPs) taking into consideration the ethical aspects of in vivo trials^6,8,9^. Up to date, different pig models have been actually proposed as suitable animal models for different eye disorders such as retinitis pigmentosa, macular degeneration, glaucoma, and cataract^10–15^. Pigs are also considered good candidates to assess biocompatibility and functionality of retinal prostheses, as well as surgical insertion procedures of the exogenous devices^16^. As for any other species and field of interest, extensive characterization and deep knowledge of animal models are pivotal when designing a study protocol. In this context, electrophysiology portrays a key functional tool to assess the results of pre-clinical ophthalmological trials. Indeed, electrophysiology represents one of the most objective ways to investigate vision providing reliable results that can support retinal imaging^17^ and behavioral analyses^18^.

Out of the various electrophysiological approaches, electroretinography is one of the most common investigation tools allowing to gain information on retinal function in both clinical and research ophthalmological settings^19^. Several types of electroretinogram (ERG) paradigms have been implemented and widely described in literature^20^, such as full field ERG (ff-ERG), multifocal ERG (mf-ERG), and pattern ERG (p-ERG). The ff-ERG provides information regarding the overall retinal function by recording a mass potential from the whole retina^21^, and has been used in biomedical pigs^5,16^. The mf-ERG can measure electrical activity from more than a hundred retinal areas per eye, allowing for more topographically precise investigations^22^. mf-ERGs have also been used and described in pre-clinical trials enrolling pig models^23,24^. The p-ERG represents a valuable tool to monitor Retinal Ganglion Cells (RGCs) activity and function^25^. It is a light-adapted response elicited by pattern stimuli^26^ that is normally used to assess visual acuity^5,27^. If accompanied by imaging and behavioral methods, the above-mentioned strategies allow for precise quantitative structure–function correlations of specific retinal regions. This experimental approach may provide important information to characterize retinal conditions and/or monitor the effects of retinal prosthesis on visual functions^16,27–30^.

Despite its potential usefulness to investigate several therapeutic approaches to retinal disorders, only scarce methodological data are available regarding the recording and interpretation of p-ERGs in biomedical pigs. The same applies to the assessment of visual acuity, which seems to have been addressed in this species only in a subjective manner^31^. Enhancing and fostering knowledge regarding pig vision physiology and its quantitative evaluation tools should allow for more robust basis for future studies, contributing to a refined and responsible use of such species. This, in turn, should lead to a high translational value of in vivo pre-clinical trials^32^. The aim of this study was to record p-ERGs in juvenile pigs to functionally assess retinal acuity.

## Results

### Optical coherence tomography

**Figure 1:**
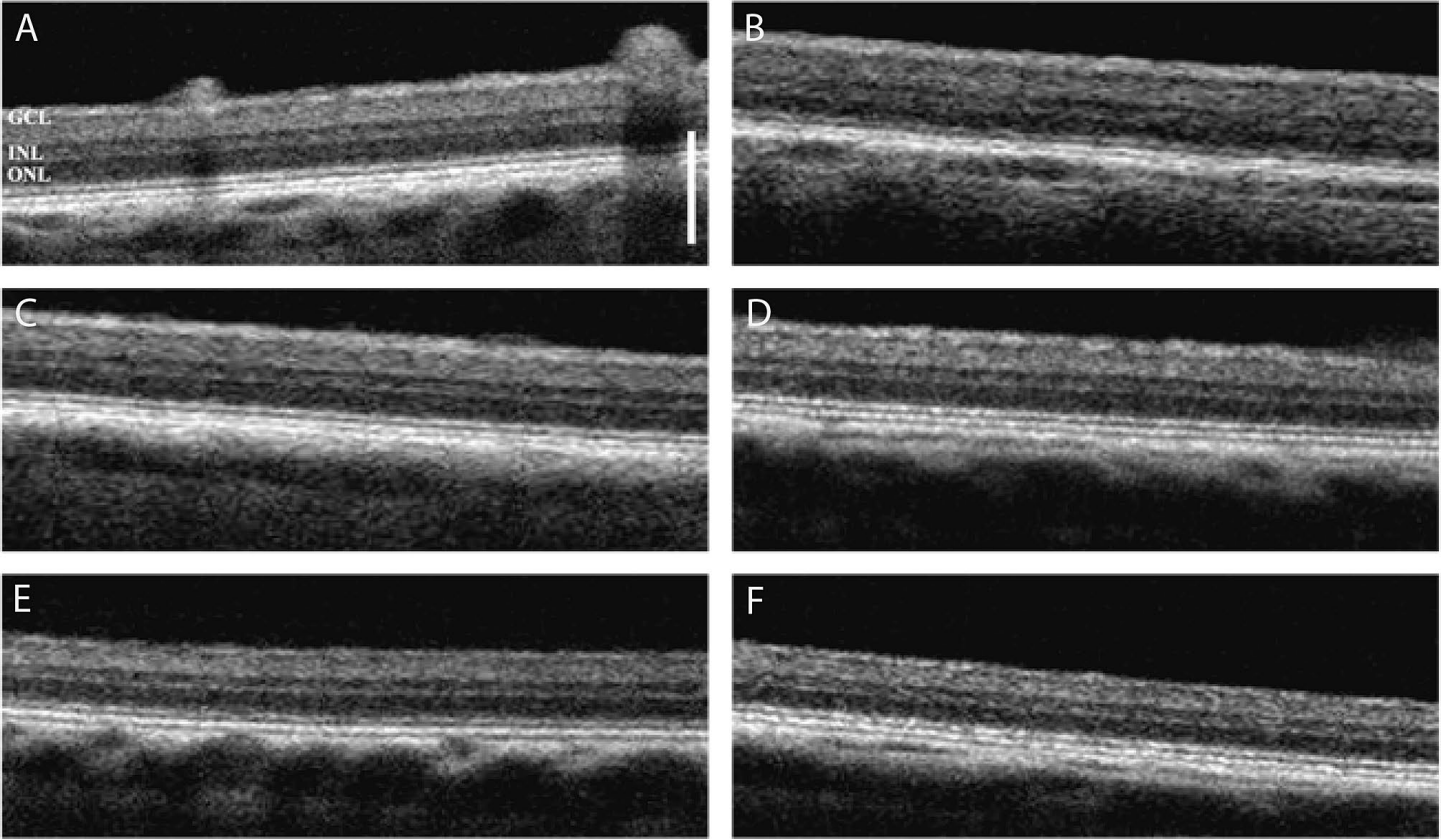
Representative optical coherence tomography (OCT) scans of the right eye in each experimental pig (**A**–**F**). The images show no morphological alterations of the retina. GCL, ganglion cell layer; INL, inner nuclear layer; ONL, outer nuclear layer. The structural organization of the retina is normal in all experimental animals. Scale bar, 250 µm.

We initially performed a spectral domain-optical coherence tomography (SD-OCT) analysis to verify the normal health state of the retina in all experimental animals. No morphological retinal abnormalities were observed as SD-OCT scans revealed full integrity (Fig. 1).

### Electrophysiology

**Figure 2:**
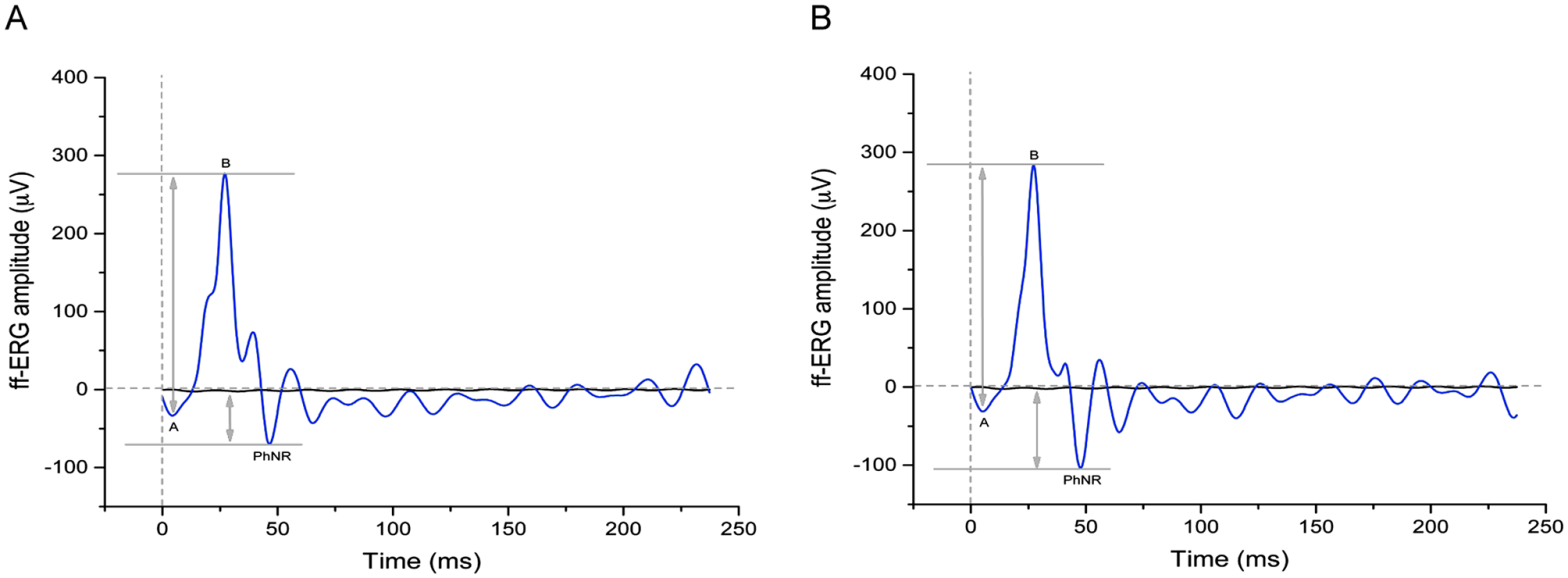
Representative ff-ERG traces for the first (**A**) and second (**B**) electrophysiological sessions. The morphology of the electrophysiological responses to flashes of light is similar but not identical between the two time points. Note that the ΔA-B response slightly increases in the second session, as does the PhNR response (midline to peak). A, a-wave; B, b-wave; PhNR, photopic negative response.

ff-ERGs were performed in each animal before shifting to p-ERGs during two recording sessions performed at 16 and 19 weeks of life. All traces (raw data) were FFT filtered to digitally eliminate the 50 Hz band (noise); representative traces are shown in Fig. 2. We found a small but significant increase (*p* = 0.0203, paired *t*-test) of light-adapted ff-ERG amplitudes (ΔA-B) between the first (237.7 ± 28.7 µV, animals *n* = 6) and the second session (272.3 ± 31.2 µV, animals *n* = 5), as expected for young animals before sexual maturation^33^. These findings suggest intact retinal functionality at the stages of development investigated, and are perfectly in line with the SD-OCT observations.

**Figure 3:**
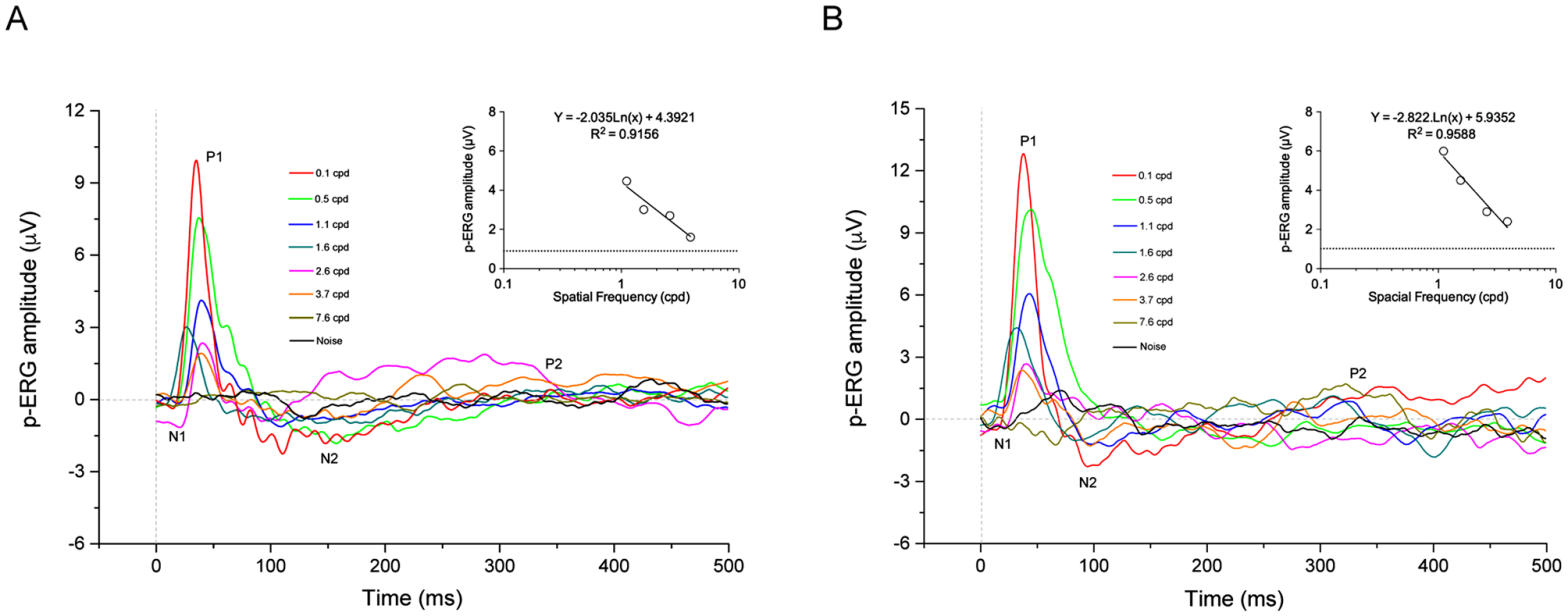
Representative p-ERG traces recorded in response to stimulation with sinusoidal gratings of increasing spatial frequency during the first (**A**) and second (**B**) electrophysiological session. The analysis of the electroretinographic responses showed the progressive decrease of p-ERG amplitude (ΔN1-P1) after enhancing the spatial frequency (different colors) of visual stimuli. Insert panels depict the regression line from which retinal acuity is computed. cpd, cycles per degree.

Because we were interested in assessing the quality of vision (i.e., retinal acuity), we next recorded transient p-ERGs in response to contrast-reversal of pattern visual stimuli while varying their spatial frequency. All traces consistently displayed peaks that characterize the electrophysiological analysis performed (*N1, P1, N2, P2*)^34,35^. The progressive decrease of p-ERG amplitude (ΔN1-P1) in response to the increment of spatial frequency of visual stimuli, in parallel with unaltered latencies of the peaks, revealed the reliability of the electrophysiological measurements in each recording session (Fig. 3).

Of note, one of the 6 animals enrolled in the present study showed severe breathing complications upon extubating after the first electrophysiology session; it was therefore re-anesthetized and euthanized (barbiturates overdose) in agreement with the previously set Humane Endpoints (HEP) for the study. For this animal, data from the first recording session were still used.

We then utilized p-ERG amplitudes, which depend on the functionality of RGCs, to compute the limit of spatial resolution in the retina (retinal acuity) in each experimental animal. The p-ERG spatial acuity was obtained by plotting the amplitude of retinal responses *versus* the logarithm of the spatial frequencies, as previously reported^36–38^. The regression line was extrapolated to the noise level. Retinal acuity was 5.7 ± 0.14 cpd in the first recording session (*n* = 6) and 6.2 ± 0.15 cpd in the second one (*n* = 5) (Table 1).

**Table 1:** p-ERG acuities calculated during both recording sessions and corresponding regression line formulas.

Animal	I Session			II Session		
	Regression line	p-ERG acuity (cpd)	noise (µV)	Regression line	p-ERG acuity (cpd)	noise (µV)
1	Y = − 2.316ln(x) + 5.3051	6.1	1.1	Y = − 2.755ln(x) + 6.2595	6.6	1.1
2	Y = − 1.808ln(x) + 4.0071	5.5	0.9	/	/	/
3	Y = − 1.392ln(x) + 2.9818	5.5	0.6	Y = − 2.822ln(x) + 5.9352	5.7	1
4	Y = − 3.234ln(x) + 6.824	5.8	1.1	Y = − 5.252ln(x) + 10.862	6.2	1.2
5	Y = − 2.035ln(x) + 4.3921	5.5	0.9	Y = − 6.659ln(x) + 13.094	6.1	1
6	Y = − 1.333ln(x) + 3.4481	5.9	1.1	Y = − 2.116ln(x) + 4.841	6.3	0.9
	Mean ± SEM	5.7 ± 0.14	1 ± 0.08	Mean ± SEM	6.2 ± 0.15	1 ± 0.04

**Figure 4:**
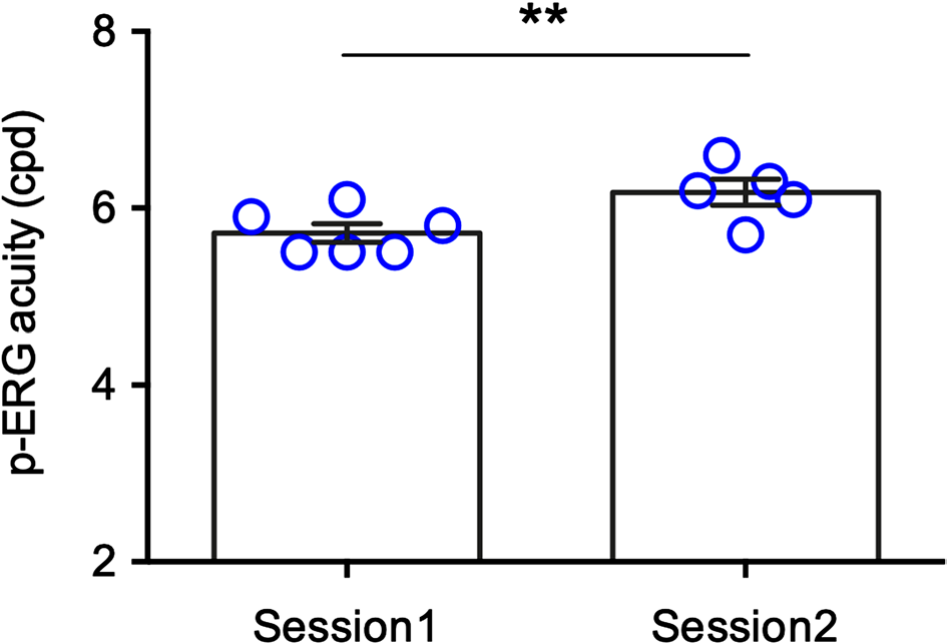
p-ERG acuity in the experimental animals during the two recording sessions. The electrical signals recorded during the first session significantly increased in the second one suggesting a developmental maturation of the spatial resolution. **p = 0.0032; paired *t*-test.

Retinal acuity was significantly lower in the first recording session (*p* = 0.0032, paired t test, p-ERG acuity) as compared to the second (Fig. 4). Interestingly, this phenomenon somewhat parallels changes of spatial acuity that occur during visual cortex development in rodents^39,40^.

**Figure 5:**
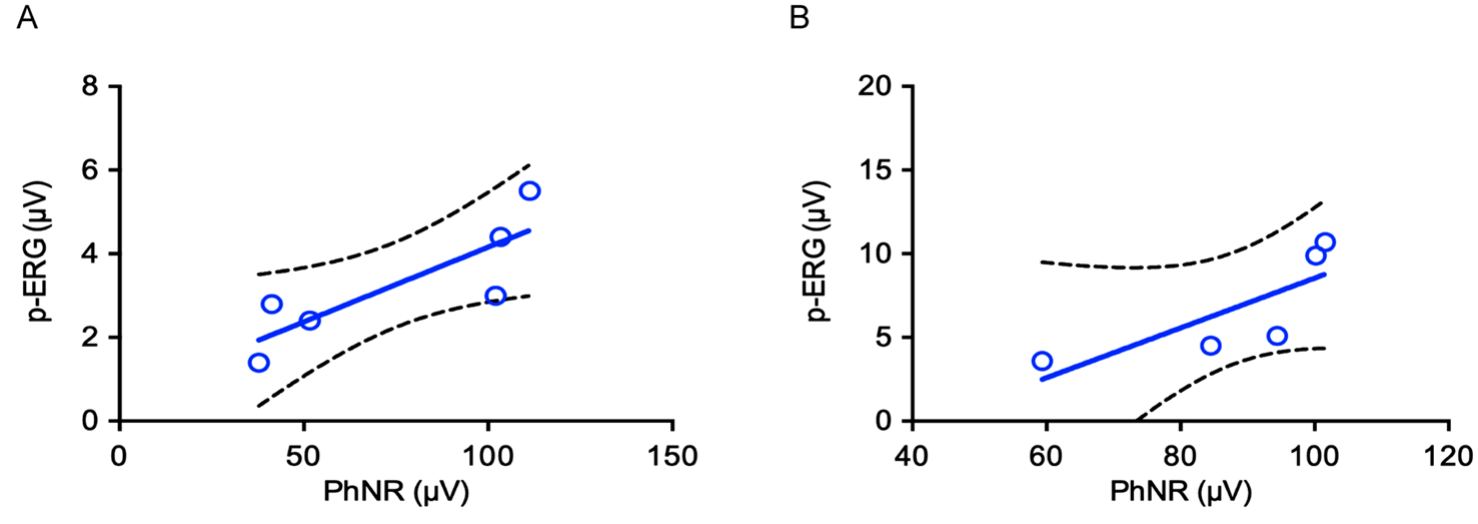
Linear regression analysis showing that p-ERG occurrence is positively correlated with the amplitude of the PhNR. The Pearson’s correlation coefficient shows a positive correlation between p-ERGs (1.552 cpd) and PhNRs during the first electrophysiological session (*p* = 0.0379). A not significant trend was observed (*p* = 0.1146) during the second session. Sample size: (**A**) *n* = 6, first session; (**B**) *n* = 5, second session.

We next addressed whether the amplitude of p-ERGs was correlated to that of the photopic negative response (PhNR) (Fig. 2; Supplementary Table 1), whose occurrence is related to the integrity of RGCs^41–43^. Remarkably, using the Pearson’s correlation coefficient, we found that the amplitude of the PhNR was positively correlated to that of the p-ERG in the first experimental sessions (r^2^ = 0.6998, *p* = 0.0379, animals *n* = 6) (Fig. 5A). We also found a positive trend, although not significant, in the second session (r^2^ = 0.6186, *p* = 0.1146, animals *n* = 5) (Fig. 5B).

### Histology

Finally, we performed an histological analysis of the dissected retinas using hematoxylin–eosin staining, as previously described^5,44^. In agreement with the SD-OCT data, histology images revealed that the retina was normally stratified and presented no pathological features (Supplementary Fig. 1). A mean nuclear count of 4.72 in the outer nuclear layer (ONL) and 3.09 in the inner nuclear layer (INL) was calculated; RGCs were 7.21 (Supplementary Table 2). Interestingly, from the central to the peripheral retina, there was a gradient of homolateral decrease of RGCs density, as previously reported^45^. The normality of cells density per retinal layer further validates the electrophysiological analysis.

## Discussion

The aim of the work hereby presented was to characterize and use the p-ERG in biomedical pigs as an additional tool for the objective assessment of vision in such species. Indeed, proposing and refining analytical procedures such as retinal electrophysiology, represents a mandatory step towards the standardization of pre-clinical trials that may lead to a reduction in the number of animals enrolled by increasing robustness of the results.

One of the pigs enrolled in the study was euthanized upon awakening from the first recording session due to severe respiratory problems that were non-compatible with the previously established HEPs. Necroscopy highlighted lungs lesions compatible with enzootic pneumonia that caused breathing issues upon extubating. Unfortunately, subclinical pneumonia is a relatively common finding when working with commercial porcine breeds^46^, and represents, aside from size and logistics issues, the biggest pitfall of using conventional commercial porcine breeds. We decided to include the data of the euthanized pig recorded during the first session, as the underlying pathology was not deemed capable of altering ERG recordings. The other animals did not show any complication and successfully completed all trials as designed.

The anesthesia protocol chosen for the study is commonly used in biomedical pigs as the combination of tiletamine-zolazepam and dexmedetomidine allows to reduce the injection volume required to achieve sedation when compared to ketamine combinations^47^. Nonetheless, more discussion is due regarding the maintenance regimen used for general anesthesia when recording ERGs. Generally speaking, inhalational anesthetics such as isoflurane and sevoflurane represent the most common choices for pigs when ventilation devices and trained equipment are available as they are considered as safe and easier to manage^47^. However, the use of halogenated anesthetics for ERGs and visually evoked potential (VEP) recording is controversial as they can affect electrical conduction and contrast sensitivity in patients^48,49^. In this context, few studies have been performed in other species to evaluate the variability in amplitude, latency and morphology of ERG traces induced by sevoflurane anesthesia^50,51^. Since related background data in pig is limited, if not absent, we decided to use a total intravenous anesthesia approach, by means of a propofol CRI (constant rate infusion), to minimize potential biases to the recording sessions. Such approach is well known and characterized in the porcine species used for biomedical purposes^52^.

To strengthen the reliability of our results, we performed both in vivo SD-OCT analyses and postmortem histological evaluations to confirm eye and retinal health status. Both approaches confirmed the physiological condition of the animals, with normal retinal stratification and cell density in the different areas. As for the p-ERG recordings, the procedure was relatively easy and fast. Data were acquired using JET disposable contact lens electrodes, chosen amongst different electrodes (conductive fibers and foils), and widely used for other ERG analyses in pigs^5,16,18^. Due to the preliminary investigative nature of the study, a wide range of spatial frequencies from 0.1 to 7.6 cpd was used, allowing for in-depth analyses of the outcoming electrophysiological traces. The overall morphology of the p-ERG waves was maintained up to 3.7 cpd, with progressively decreasing amplitudes starting from 0.1 cpd. These findings indicate that our experimental conditions are reliable and allow for correct p-ERG recording and analysis. In our hands, data recorded in response to 7.6 cpd spatial frequency did not differ from the noise band. Because the eyes of the animals were not refracted, the possibility arises that p-ERG acuity measurements could have been affected by blurring. Further analysis of retinal acuity using spatial frequencies between 3.7 and 7.6 cpd, however, should be focus of attention in future experiments. As stated before, the amplitudes of the p-ERGs statistically differed between the first and the second recording session, with the latter being higher. Such difference may be explained by the age of the animals, almost at the end of visual cortex development. A similar finding has indeed been described in the developing visual cortex of rodents^39^. To the best of our knowledge, few electrophysiological data are available regarding the maturation of the visual system in pigs, but a general consensus exists that large breed can be considered adults after the 5th–6th month of life with intersexual differences^33^. Considering this, the two recording sessions, performed at 16 and 19 weeks of age, depict a dynamic developmental phase of the pig visual system.

ff-ERG recordings were included in the experimental design to validate the interpretation of the p-ERG finding. Not only they provide general information regarding the whole retina health status, but one specific component, the photopic negative response (PhNR), is directly correlated to RGCs integrity^41–43^. Since p-ERG reflects RGC functionality, we performed a correlation analysis between PhNR and p-ERG amplitude to validate our results. A significant positive correlation was found in the first recording session, while the second one showed a positive correlation trend, likely due to the loss of one animal after the first trial. Despite further investigations being needed, ff-ERG confirmed the validity of our p-ERG recordings.

We used p-ERG amplitudes to compute the limit of spatial resolution at retinal level in each experimental pig. Despite this issue deserving further investigations, we observed a higher retinal acuity during the second recording session at 19 weeks of age when compared to the ones recorded at 16 weeks. When looking at the literature to compare our results, it emerges that visual acuity in pigs has been barely investigated^31^, often with different methodological approaches, such as Landlord C symbols, making a direct comparison almost impossible. Mean p-ERG acuity values recorded during the two sessions were 5.7 ± 0.14 cpd for the first one and 6.2 ± 0.15 cpd for the second. Both values are lower when compared to visual acuities in humans (~ 30 cpd) and rhesus monkeys (~ 23.7 cpd)^53^, but much higher than C57BL/6 J mice (~ 0.6 cpd)^26^ and rats (~ 1 cpd)^38^. It is nonetheless interesting to note that the retinal acuity values we report in the present study somewhat approach the ones recorded in two other important large animal models such as Beagle dogs^54^ and cats^55^.

Overall, the p-ERG recordings described in the present work seem to be reliable and repeatable, as supported by the data validations performed a posteriori. This may represent an important tool to add to the already available ones when it comes to vision assessment in pigs. As this species gains ground in the field of experimental and translational ophthalmology, providing the scientific community with new analytical methods, it represents a relevant possibility for the refinement of experimental procedures and conduction of research. The ability to record reliable p-ERG traces and quantify spatial discrimination in pigs may be of great help in assessing visual restoration after surgical implantation of retinal prostheses.

## Methods

### Animals

The study was performed on six (N = 6) female commercial hybrid pigs [(Large White × Landrace) × Duroc] from the same litter, born and housed in the experimental porcine facility of the Department of Veterinary Medical Sciences of the University of Bologna. At the time of the first electrophysiology session, animals were 16 weeks old with a mean weight of 24.7 kg (SD: 4.0 kg), while at the second recording session they were 19 weeks old with a mean weight of 27.6 kg (SD: 5.3 kg). The six animals were housed in two multiple pens, with a light/dark cycle of 12:12 h (minimum of 50 lx during light periods), and a temperature of 22 ± 1 °C; pigs were fed a commercial standard diet, with free access to water, and in the presence of dedicated plastic environmental enrichments (Porcichew, Best Balls and Superchallengers; Plexx B.V., ELST, NL) provided to minimize stress. Animals were daily trained and accustomed, by means of food rewards as positive reinforcement, to interact with humans. From a microbiological point of view, the facility is officially Pseudorabies- and SVD-free, and animals tested negative for PRRS, PPV and PCV.

All procedures were performed according to the Statement for the Use of Animals in Ophthalmic and Vision Research of the Association for Research in Visual Ophthalmology (ARVO) and in compliance with the ARRIVE (Animal Research: Reporting of In Vivo Experiments) guidelines.

The experimental protocol was approved by the Animal Welfare Body of the University of Bologna first, and then by the Italian Ministry of Health (Ministero della Salute) as dictated by D.Lgs 26/2014 (n. 887/2016-PR).

### Anesthesia

On recording days, animals were weighted and sedated with an intramuscular (IM) injection of a mixture of tiletamine-zolazepam (3 mg/kg, Zoletil; Virbac, Prague, CZ) and dexmedetomidine (0.02 mg/kg, Sedadex; Dechra, Torino, IT) behind the base of the ear, upon 12 h fasting. Once injected, animals were left in a dark and noise-free environment, under strict veterinary monitoring, for 15 min. A peripheral vascular access was achieved through an auricular vein and used to induce general anesthesia by means of an IV propofol bolus (2–3 mg/kg, Proposure; Merial, FR), and to grant fluid therapy (lactated ringer 6 ml/kg/h) throughout the entire procedure. Immediately after induction, a propofol constant rate infusion (CRI, 0.1–0.2 mg/kg/min) was started to maintain general anesthesia. Animals were placed in sternal recumbency, orotracheally intubated with a Murphy cuffed tube, and mechanically ventilated (PVP mode set at 12 mmHg, respiratory rate adjusted to maintain normocapnia) upon a single dose of atracurium besylate (1 mg/kg, Tracrium; GSK, Brentford, UK). Temperature was maintained within the physiological range using a Bair Hugger normothermia system (3 M, Minnesota, USA).

Each recording session, from sedation to standing up, lasted approximately 2 h. Upon electrodes removal, eyes were washed with sterile saline and an antibiotic ointment (Colbiocin; SiFi s.p.a., Catania, IT) was applied on the conjunctiva. Except for the single animal euthanized after the first recording session due to respiratory complications, all animals completely recovered from anesthesia within a maximum of two hours, to be moved back to their origin pen.

### Spectral domain optical coherence tomography (SD-OCT)

All procedures herein described, including electrophysiological recordings, were performed on the right eye of each animal to reduce the duration of the analyses sessions.

Animals underwent an SD-OCT analysis using the OptoVue iVue 100 (software iVue V3.2; Optovue Inc., Fremont CA, US), using 840 ± 10 nm light with power at the pupil of 750 μW. The depth of resolution in tissue is 5 μm, and the transverse resolution is 15 μm. Each image covered a 6 × 6 mm area centered on the fovea, acquired at 26.000 A-scan/second and composed of 256 to 1024 A-scan/frame^16^. Pupils were dilated with 1 drop of tropicamide 10%/phenylephrine 0.5% eye drops (Visumidriatic Fenilefrina; Visufarma, Roma, IT), and animals were placed in sternal recumbency with the right side of the face. Local analgesia was provided by means of 2 drops of oxybuprocaine hydrochloride (Novesina; Visufarma s.r.l., Rome, IT) onto the corneal surfaces, then a sterile Barraquer blepharostat was placed. Throughout the scanning procedure, corneal hydration was granted by administration of hyaluronic acid artificial tears (Hyalistil 0.2%; SIFI s.p.a., Catania, IT).

### Electrophysiology

For electrophysiology recordings, corneal disposable contact lens electrode (ERG-jet, Universo Plastique, Switzerland) with a drop of benzalconium chloride polyacrylic acid gel (Lacrinorm, FARMIGEA Holding S.r.l., Pisa, IT) were used as active electrode with dermal needle electrodes used as reference placed under the ipsilateral eyelid and aborally on the snout. All electrophysiological data were acquired and amplified using the Retimax system (C.S.O. srl, Florence, Italy) as previously reported^5,18^. The ff-ERG stimuli were produced by a MiniGanzfeld device; 100 sweeps were averaged with a band pass filter between 1 and 100 Hz, 3 k gain, and an acquisition time of 250 ms. Stimuli were flashes of light (3 cd/s/m^2^) at 1 Hz frequency. Light adaptation was of 20 min (30 cd/m^2^). The p-ERG stimuli, instead, were produced by a 17 inches 1024 × 768 resolution screen connected to a pattern generator. Stimuli were alternating white and black vertical gratings (squarewave) of 98% contrast, with a constant temporal frequency of 2 rps (0.5 Hz) and reverse temporal form. The distance between the screen and the eye was set at 30 cm, with 59 degree visual angle. The background luminance was 50 cd/m^2^. For each p-ERG analysis, 200 sweeps were averaged with a band pass filter between 1 and 30 Hz, 30 k gain, and acquisition times of 500 ms. Different spatial frequencies, expressed as cycles per degree of visual angle (cpd), were presented: 0.101, 0.517, 1.109, 1.552, 2.587, 3.88, and 7.61 cpd. As a control for all recordings, visual stimuli with 0% contrast were used. Peak detection was set above three-fold the standard deviation of the basal electrical signal^25^. The electrical impedance for all electrodes was lower than 5 kΩ, while artifact signals were automatically recognized and excluded by the software. In each electroretinogram peak-to-peak amplitudes (ΔA-B for ff-ERG; ΔN1-P1 for p-ERG) and midline to peak (for the PhNR of the ff-ERG) were expressed in microvolts (μV) and retention times (latencies) in milliseconds (ms). Retinal acuity was obtained by plotting the amplitude of retinal responses versus the logarithm of the spatial frequency, as previously reported^35,36,53^.

### Histology

The right ocular globes were fixed in 10% formalin and trimmed with a sagittal section perpendicular to the long posterior ciliary artery and adjacent to the optic nerve. Three serial section 4 µm-thick for each ocular globe were obtained from paraffin-embedded tissue and routinely stained with hematoxylin and eosin.

The nuclear profile, for both the outer and inner nuclear layers, was evaluated according to Scott et al.^44^.

Ten adjacent columns of nuclei were consecutively counted in three serial sections and for each section in the following four topographical points: (1) visual streak, dorsal (dorsal, 2 mm from the optic nerve head); (2) dorsal periphery (dorsal, 8 mm from the optic nerve head); (3) ventral central (ventral, 2 mm from the optic nerve head); (4) ventral periphery (ventral, 8 mm from the optic nerve head). Retinal ganglion cells density was counted in each of the mentioned topographical points, according to a previous reported method^5^.

### Data analysis

All electrophysiological data was analyzed using the OriginPro software (OriginPro, 93E version, OriginLab Corporation, Northampton, MA, USA). A digital 50 Hz band-block FFT filter was used for all electrophysiological traces to eliminate noise. For ff-ERG traces, the amplitude from A to B peaks was measured; for p-ERG traces we measured the N1 to P1 amplitudes; for PhNR we measured the N2 to baseline amplitudes. As for histology, normality of the data was assessed according with D’Agostino and Pearson normality test (GraphPad Prism, Version 9, GraphPad Software, San Diego, CA, USA).

## Supplementary Information

Supplementary Information.

## Data Availability

The data of this manuscript are available from the corresponding author upon reasonable request.
