# Supplementary material for: The p-ERG spatial acuity in the biomedical pig under physiological conditions
Source: Sci Rep. 2022 Sep 14;12:15479. doi: 10.1038/s41598-022-19925-8 (PMC9474814; doi:10.1038/s41598-022-19925-8)
Supplement: Supplementary file 1 — Supplementary Information. [file 41598_2022_19925_MOESM1_ESM.docx]

# SUPPLEMENTARY MATERIAL

# The p-ERG spatial acuity in the biomedical pig under physiological conditions

Domenico Ventrella, José Fernando Maya-Vetencourt, Alberto Elmi, Francesca Barone, Camilla Aniballi, Luisa Vera Muscatello, Maurizio Mete, Grazia Pertile, Fabio Benfenati, Maria Laura Bacci

**Supplementary Figure 1. Normally stratified retina, hematoxylin and eosin, 400x.** NFL, nerve fiber layer; GCL, ganglion cell layer; IPL, inner plexiform layer; INL, inner nuclear layer; OPL, outer plexiform layer; ONL, outer nuclear layer; PRL, photoreceptor layer; RPE, retinal pigmented epithelium.

**Supplementary Table 1.** Individual amplitudes of the ff-ERG photopic negative response (PhNR).

| **Animal** | **PhNR (µV)** | |
| --- | --- | --- |
|  | **Session 1** | **Session 2** |
| 1 | 103.4 | 94.4 |
| 2 | 37.8 | / |
| 3 | 51.6 | 84.54 |
| 4 | 102 | 100.2 |
| 5 | 111.3 | 101.6 |
| 6 | 41.3 | 59.4 |

**Supplementary Table 2.** Average count of the nuclei in the three analyzed retinal layers in four topographical points. Data are reported as mean ± S.E.M. ONL, outer nuclear layer; INL, inner nuclear layer; GCL, ganglion cell layer.

| **Area** | **ONL** | **INL** | **GCL** |
| --- | --- | --- | --- |
| **2mm V** | 4.47 ±0.13 | 3.04 ±0.12 | 10.83 ±2.80 |
| **8mm V** | 4.57 ±0.23 | 3.08 ±0.19 | 6.50 ±1.20 |
| **2mm D** | 5.28 ±0.08 | 3.36 ±0.23 | 7.83 ±1.97 |
| **8mm D** | 4.56 ±0.31 | 2.87 ±0.18 | 3.67 ±1.52 |
